# The Small GTPase Cdc42 Negatively Regulates the Formation of Neutrophil Extracellular Traps by Engaging Mitochondria

**DOI:** 10.3389/fimmu.2021.564720

**Published:** 2021-02-17

**Authors:** Heidi Tackenberg, Sonja Möller, Marie-Dominique Filippi, Tamás Laskay

**Affiliations:** ^1^Department of Infectious Diseases and Microbiology, University of Lübeck, Lübeck, Germany; ^2^Division of Experimental Hematology and Cancer Biology, Cincinnati Children's Hospital Medical Center and University of Cincinnati College of Medicine, Cincinnati, OH, United States

**Keywords:** small GTPases, Cdc42, neutrophil granulocytes, neutrophil extracellular traps, mitochondria, mitochondrial ROS, mitochondrial membrane potential

## Abstract

Neutrophil granulocytes represent the first line of defense against invading pathogens. In addition to the production of Reactive Oxygen Species, degranulation, and phagocytosis, these specialized cells are able to extrude Neutrophil Extracellular Traps. Extensive work was done to elucidate the mechanism of this special form of cell death. However, the exact mechanisms are still not fully uncovered. Here we demonstrate that the small GTPase Cdc42 is a negative regulator of NET formation in primary human and murine neutrophils. We present a functional role for Cdc42 activity in NET formation that differs from the already described NETosis pathways. We show that Cdc42 deficiency induces NETs independent of the NADPH-oxidase but dependent on protein kinase C. Furthermore, we demonstrate that Cdc42 deficiency induces NETosis through activation of SK-channels and that mitochondria play a crucial role in this process. Our data therefore suggests a mechanistic role for Cdc42 activity in primary human neutrophils, and identify Cdc42 activity as a target to modulate the formation of Neutrophil Extracellular Traps.

## Introduction

Since the discovery of a novel cell death mechanism in 1996 that was different from apoptosis or necrosis, considerable effort was made to further characterize this phenomenon (1). In 2004, Brinkmann also reported a form of neutrophil cell death, leading to DNA extrusion during bacterial infection (2). This novel form of programmed cell death was termed NETosis in 2007 (3). Since the first description of the formation of Neutrophil Extracellular Traps (NETs) it was demonstrated that various stimuli, such as lipopolysaccharides (LPS), interleukin 8 (IL-8), and tumor necrosis factor alpha (TNFα) but also pathogens can induce the extrusion of DNA in neutrophil granulocytes (4). Although originally discovered in polymorphonuclear cells (PMN), it is now clear that other granular cell types such as eosinophils and mast cells undergo a NETosis like process (5). NETs are composed of chromosomal DNA that is associated with nuclear histones, granular antimicrobial proteins, and some cytoplasmic proteins (6–10). The extruded DNA appears as a web like structure that is made of long DNA fibers, perfectly suitable to bind and trap pathogens. The released DNA structures occupy three to five times the volume of condensed chromatin (1). Diverse proteins adhere to the DNA, including histones as well as over 30 components of primary- and secondary granules (6–10). Among these granular components are elastase, myeloperoxidase (MPO), cathepsin G, lactoferrin, proteinase 3 (PR3), and others with bactericidal functions (6–10). Recently it was shown that NETs can also contain mitochondrial DNA (11). Although NOX complex-assembly and subsequent ROS release was originally reported to be essential for the induction of NETosis, the precise role of ROS in NETosis has become a topic of controversy (5). This controversy arises due to the observation that stimulation of neutrophils with N-formylmethionine-leucyl-phenylalanine (fMLP), a potent inducer of NOX activity, was not able to induce NETs (5). In contrast, LPS and IL-8 which were reported to induce NETs are not triggering NOX activity (12, 13). Additionally, it was shown that calcium ionophores like ionomycin induce NET formation in an NOX-independent pathway. The results of Lood et al. suggested that mitochondrial ROS (mtROS) synthesis is sufficient to induce NETs without the activity of NOX (11). This phenomenon might promote the formation of NETs enriched in mitochondrial DNA in chronic granulomatous disease (CGD) and systemic lupus erythematosus (SLE) patients (11).

Because of the afore-mentioned observations, the existence of two main pathways for NET formation is appreciated. The NOX-dependent NET formation has PMA as its model inducer and requires the formation of ROS. The NOX-independent pathway on the other hand is independent on NOX-generated ROS. However, the major regulators of this NETosis pathway are still largely unknown (4). Although NET formation is an antimicrobial response that should be beneficial for the host, it is known that they are also major contributors to different pathologies, especially autoimmune rheumatic diseases (ARDs) like rheumatoid arthritis (RA) and SLE (1).

Small GTPases (sGTPases) of the Rho-subfamily are essential for neutrophil granulocyte migration to sites of infection by controlling cytoskeletal rearrangements (14, 15). Small GTPases are molecular switches cycling between the active GTP-bound and the inactive GDP-bound state (14). GTP exchange factors (GEFs) and GTPase activating proteins (GAPs) enable this cycling (14). Small GTPases exist in their inactive state in resting cells. Upon stimulation with cytokines, chemokines or growth factors, different Rho-specific guanine nucleotide exchange factors (Rho-GEFs) are activated (14). Additionally to the GDP/GTP-exchange, sGTPases are regulated by their subcellular location and require docking onto the cell membrane to exert their cellular functions (14). Once activated and relocated, they interact with several effectors, enabling the engagement of specific signaling cascades (16). The sGTPase Cdc42 is a known key regulator of actin and tubulin reorganization, cell-cell and cell-extracellular matrix adhesion as well as cell polarity in different cell types (14, 17, 18). Cdc42 is localized to the leading edge upon chemoattractant stimulation and controls directed migration in several cell types (16). Another member of the Rho GTPase family, Rac, is known to play a pivotal role in the formation of ROS and cell motility (19–22). Cdc42 and Rac share some GEFs but influence migration differently. This suggests that both sGTPases share some signaling pathways while having distinct subsets of downstream targets. This would mean that not only Rac but also Cdc42 plays a special role for other neutrophil effector mechanisms like NETosis.

In the present study, we aimed to elucidate the role of Cdc42 activity in the regulation of NET formation in neutrophil granulocytes.

## Materials and Methods

### Isolation of Primary Human Neutrophils

Primary human neutrophils were isolated from peripheral blood of healthy volunteers by Histopaque and Percoll gradient centrifugation as described elsewhere (23). The blood collection was conducted with the understanding and consent of each participant and was approved by the ethical committee of the Medical Faculty of the University of Lübeck (18–187). Blood was layered on top of a density gradient consisting of Histopaque 1119 and 1077 (both from Sigma Aldrich, St. Louis, MO, USA) and centrifuged 5 min, 54 × g and further 25 min 216 × g at room temperature (RT). Granulocytes were collected and added on top of a Percoll gradient consisting of 65–85% Percoll (GE Healthcare, Buckinghamshire, UK). Following centrifugation for 30 min, 863 × g, granulocytes were collected. The cell preparations contained >99% granulocytes as determined by morphological examination of Giemsa-stained cytocentrifuged slides (Shandon, Pittsburgh, PA). PMNs were cultured in complete medium; RPMI1640 medium supplemented with 10 mM HEPES, 10% heat inactivated fetal bovine serum (Sigma Aldrich, St. Louis, MO, USA), 4 mM L-glutamine (Biochrom, Berlin, Germany).

### Mice and Isolation of Murine Neutrophil Granulocytes From Bone Marrow

Murine neutrophils were isolated from bone marrow of Cdc42^fl/fl^ and Cdc42^Δ/Δ^ mice. The use of conventional Cdc42 gene-targeted mice, carrying a whole-body knockout of Cdc42, is not possible, since these mice die at the embryonic day 7.5 (24). Therefore, the use of a conditional knock out of Cdc42 in mice was used. Cdc42^loxP/loxP^ floxed mice possess *loxP* sites, flanking exon 2 of the cell division cycle 42 (*Cdc42*) gene (25, 26). Homozygotes are viable and fertile, here referred to as Cdc42^fl/fl^ mice. When bred to mice that express tissue-specific Cre recombinase, resulting offspring will have exon 2 deleted in the *cre*-expressing tissues. Cdc42^loxP/loxP^ mice were crossbred with transgenic Myxovirus resistance-1 (Mx1)-Cre mice to allow interferon (IFN)-inducible Cdc42 gene excision in hematopoietic cells (24, 25), these mice were here referred to as Cdc42^Δ/Δ^ mice. Mx1 is a vital part of viral defense mechanisms and its expression can be highly induced in response to IFN (27). The Cre-recombinase under control of the Mx1-promoter can be activated by mimicking a viral infection (27). This is achieved by inducing a type-I IFN response through administration of synthetic double stranded RNA poly (I:C) (27). At 5 days after the administration of three doses of poly (I:C) to induce an IFN response in Mx1-Cre:Cdc42^loxP/loxP^ mice (Cdc42^Δ/Δ^), the total cellularity of bone marrow did not change but the floxed Cdc42 gene sequences and Cdc42 protein became undetectable in bone marrow cells (24, 26). Bone marrow was isolated as described elsewhere (28). Briefly, after flushing all bones into the same tube, containing 2 mL 1 × HBSS (supplemented with 0.01% BSA, w/o MgCl_2_, w/o CaCl_2_), BM cells were mixed thoroughly and filtered through a cell strainer. This was followed by washing the cell suspension for 10 min, 863 × g at 4°C and subsequent re-suspension of the cell pellet in 3 mL 45% Percoll^TM^. A density gradient consisting of five layers with differently concentrated Percoll^TM^, ranging from 81 to 45% was prepared. The density gradient was prepared by gently layering the different Percoll^TM^ concentrations on top of each other, starting with 3 mL of the 81% dilution. Two milliliters of the 62%-, 55%-, and 50% dilutions were layered on top of the 81% dilution. The previously prepared whole BM cell suspension in 45% Percoll^TM^ was layered on top of the gradient, followed by centrifugation for 30 min, 733 × g, w/o brake at 10°C. After centrifugation, a typical cell distribution pattern within the density gradient could be observed. The upper ring contains remaining BM-myeloid precursor cells, while mostly granulocytes can be found in the lower separation layer. This ring was carefully collected in a falcon tube, and washed for 10 min, 863 × g, 4°C with 1 × HBSS (supplemented with 0.01% BSA, w/o MgCl_2_, w/o CaCl_2_). In order to remove most of the erythrocytes contaminating the granulocyte fraction, the cells were re-suspended in 3 mL BM-isolation medium and layered on top of 3 mL Histopaque 1119. After centrifugation for 20 min, 863 × g at RT w/o brake, granulocytes appeared at the interface of Histopaque 1119, and 1 × HBSS (supplemented with 0.01% BSA, w/o MgCl_2_, w/o CaCl_2_). The granulocytes were collected into a falcon tube, washed for 10 min, 863 × g and re-suspended in 2 mL BM-isolation medium. The cell number of BM granulocytes was determined using the Hemavet 950FS Hemocytometer (Hemavet 950FS, Miami Lakes, FL, USA).

### Induction and Detection of Neutrophil Extracellular Traps (NETs)

Staining with the non-cell-permeable DNA intercalating dye SYTOX green (Invitrogen, Eugene, USA) was used to study the kinetics of NET formation by PMN, incubated with or without the Cdc42 inhibitor casin (10 μM) (TOCRIS, Wiesbaden, Germany). Freshly isolated neutrophils (2 × 10^5^ per well) were diluted in NET medium (RPMI 1640 supplemented with 10 mM HEPES buffer (PAA, Pasching, Austria) and 4 mM L-glutamine (Biochrom, Berlin, Germany) and seeded to a cellstar 96-well black-fluotrac plate (Greiner Bio-One, Frickenhausen, Germany). To detect the extracellular DNA of NETs, 5 μM SYTOX green was added, and neutrophils were stimulated with 20 nM PMA, 10 μM ionomycin (all from Sigma Aldrich, St. Louis, MO, USA) or the Cdc42 inhibitor casin was added (10 μM). To analyze the role of calcium in NET formation, freshly isolated human neutrophil granulocytes were re-suspended in 1 × HBSS supplemented with 10 mM HEPES, 4 mM L-glutamine (Biochrom) either with or without 1.26 mM CaCl_2_. The fluorescence of NET bound SYTOX green (excitation: 488 nm, emission: 510 nm) was analyzed for a period of 4 h every 5 min at 37°C using an infinite 200 reader and Tecan i-control 1.7 Software (Tecan).

Additionally, primary human (1.5 × 10^6^ per sample) or bone marrow derived murine (2 × 10^6^ per sample) neutrophils were seeded into a 24-well plate in NET medium or 1 × HBSS, supplemented with 0.01% BSA, 2 mM CaCl_2_ and 2 mM MgCl_2_. NET formation by human neutrophils was induced by adding 20 nM PMA, 10 μM ionomycin or 10 μM casin. NET formation by bone marrow derived murine neutrophils was induced after priming of cells with 20 ng/ml TNFα (Peprotech, Rocky Hill, NJ, USA), for 20 min, by adding 100 nM PMA, 5 μM ionomycin, or 10 μM casin. After 4 h incubation at 37°C, cells were collected and centrifuged for 10 min, 863 × g, RT. Supernatants were collected and subjected to an MPO-DNA ELISA (human cells, Order No.:11774425001 Sigma Aldrich, St. Louis, MO, USA) or an Histone H1, H2A, H2B, H3, H4-DNA-ELISA (mouse cells, Order No.:11544675001 Sigma Aldrich, St. Louis, MO, USA). Prior to the experiment, a Maxisorp 96-well plate was coated with the anti-MPO or anti-histone H2A, H2B, H3, H4 capture antibody, respectively, diluted in bicarbonate buffer, and incubated at 4°C, overnight. On the day of the experiment, wells were washed 3 times with 200 μl washing buffer followed by 30 min blocking with blocking buffer at RT. Thereafter, cell culture supernatants were added to the wells and incubated for 2 h at RT, while gently shaking. After incubation, wells were washed 3 times with 200 μl wash buffer, followed by incubation with a peroxidase-conjugated anti-DNA detection antibody for 2 h at RT. Thereafter, wells were washed 3 times with 200 μl wash buffer. Subsequently, 100 μl of ABTS substrate solution was applied to each well, and incubated for 20 min at RT while gently shaking (250 rpm). To stop the reaction, 100 μl of ABTS stop solution (part of the ELISA kit) was added per well, followed by measuring the optical absorbance at 405 nm (DNA detection) using a reference wavelength of 492 nm.

### Microscopical Assessment of NET Formation

To visualize NETs, fluorescence microscopy was performed. Freshly isolated neutrophils (5 × 10^5^) were centrifuged 10 min, 863 × g, and re-suspended in NET medium. Cells were seeded onto Poly-L-lysine-coated cover slips (BD BioCoat Cellware, Bedford, USA) and incubated with 20 nM PMA or 10 μM casin for 4 h at 37°C. Untreated samples were used as control.

After the incubation, cells were fixed with 4% PFA (Sigma Aldrich, St. Louis, MO, USA) for 15 min at RT. Subsequently, the supernatant was removed and the air-dried coverslips were rehydrated with PBS and stained with 5 μM SYTOX green for 30 min in the dark at RT. Following washing three times with PBS, the samples were mounted with ProLong GOLD antifade reagent (Invitrogen) and analyzed using a BZ-9000-E Fluorescence Microscope (Keyence Co., Osaka, Japan).

### Inhibitors to Characterize NET Formation

To study the influence of Cdc42 on the formation of NETs, the Cdc42 activity-specific inhibitor (casin) was used (TOCRIS, Wiesbaden, Germany). Casin is a Pir1 analog, and confers the most active Cdc42 binding activity without binding to related RhoGTPases (18, 29, 30). Casin was shown to specifically bind to Cdc42, and to competitively interfere with its guanine nucleotide exchange (GEF) activity, therefore, suppressing Cdc42-GTP formation (29). Inhibition of Cdc42 with 10 μM casin was shown to be most efficient to reduce cell adhesion and migration, without affecting Rac1-GTP formation. Casin is superior to other Cdc42 inhibitors, such as ML141 or AZA1, since these inhibitors also target other Rho GTPases such as Rac1 or RhoA (18, 29, 30). Therefore, no other highly selective Cdc42 inhibitor is available. To elucidate the influence of various signaling pathways in the Cdc42 dependent NET formation, specific inhibitors were used. To analyze the influence of the NADPH-oxidase, the inhibitors Diphenyleneiodonium chloride (DPI, 20 μM) and VAS2870 (20 μM) (both from Sigma Aldrich, St. Louis, MO, USA) were used. For the determination of the role of Protein kinase C in NET formation, the specific inhibitor Dihydrosphingosine (DHS) (4 μM) was used (Abcam, Cambridge, UK). The influence of small conductance calcium-activated potassium channels (SK-channels) and the protein arginine deiminase 4 (PAD4) on NET formation was analyzed using the specific inhibitors NS8593 (100 μM) (TOCRIS bioscience, Bristol, UK) and Cl-amidine (10 μM) (Sigma Aldrich, St. Louis, MO, USA), respectively. The role of mitochondrial Reactive Oxygen Species in NET formation was analyzed using the mitochondrial ROS inhibitor mitoTEMPO (20 μM) (Thermo Fisher Scientific, Waltham, MA, USA) and the mitochondrial respiratory chain complex I and III inhibitors rotenone (1 μM) (Sigma Aldrich, St. Louis, MO, USA, Cat. No. A8674) and antimycin A (1 μM) (Sigma Aldrich, St. Louis, MO, USA, Cat. No. R8875).

### Detection of Mitochondrial ROS Formation Using Live Cell Imaging and Flow Cytometry

Sixty thousand freshly isolated bone marrow derived murine neutrophils were seeded into a 96-well glass bottom plate per well in 1 × HBSS, supplemented with 0.01% BSA, w/o CaCl_2_, w/o MgCl_2_ and allowed to settle for 45 min at 37°C, following priming with 20 ng/ml TNFα (Peprotech, Rocky Hill, NJ, USA) for 20 min at 37°C. Afterwards, supernatants were removed and 5 μM of the mitochondrial ROS indicator MitoSOX (Thermo Fisher Scientific) was added followed by incubation for 30 min at 37°C. Supernatants were removed and cells were left untreated, or stimulated with 100 nM PMA, 5 μM ionomycin or 10 μM casin in 1 × HBSS supplemented with 0.01% BSA, 2 mM CaCl_2_, 2 mM MgCl_2_. The generation of mitochondrial ROS over time was detected using the Incucyte® Live-Cell Analysis System (Essen BioScience) for 1 h at 37°C every 4 min. The amount of mitochondrial ROS produced over time was analyzed using the IncuCyte Zoom elements 2000 software. To detect mitochondrial ROS formation in primary human neutrophils, 5 × 10^5^ freshly isolated neutrophils per sample were pre-incubated with 5 μM MitoSOX for 30 min. at 37°C. Cells were left untreated or incubated with PMA (20 nM), ionomycin (10 μM) or the Cdc42 inhibitor casin (10 μM) for 1 h at 37°C. The fluorescence signal of MitoSOX was analyzed using flow cytometry.

### Detection of the Mitochondrial Membrane Potential

5 × 10^5^ freshly isolated primary human or bone marrow derived murine neutrophils were incubated with ionomycin (5 μM mouse, 10 μM human), PMA (20 nM human, 100 nM mouse), or the Cdc42 inhibitor casin (10 μM) for 1 h at 37°C. Following centrifugation for 10 min, 863 × g at RT, cells were re-suspended in 2% BSA/PBS and stained with CD11b-FITC (human) (Merck Millipore, Billerica, Massachusetts, USA) or Ly6G-FITC (mouse) (Bio Legend, San Diego, California, USA) in a 1:100 dilution for 20 min at RT. Following centrifugation for 10 min, 863 × g at 4°C, cells were re-suspended in PBS and incubated with 200 nM Tetramethylrhodamine, Ethyl Ester, Perchlorate (TMRE) (Thermo Fisher Scientific) for 30 min at 37°C in the dark. TMRE is a positively charged, cell permeable dye that specifically binds to active, negatively charged mitochondria (31, 32). Afterwards, warm PBS was added and cells were centrifuged for 10 min, 863 × g at 4°C followed by addition of 2 %BSA/PBS and subsequent analysis by flow cytometry.

### Western Blot Analysis of Citrullinated Histones

Neutrophils (5 × 10^6^) were left untreated (control) or incubated with PMA (20 nM), ionomycin (10 μM) or with the Cdc42 inhibitor casin (10 μM) for 10 min at 37°C. Subsequently, cells were lysed with ice-cold 10% TCA (Sigma Aldrich, St. Louis, MO, USA) and incubated for 10 min on ice. Thereafter, lysates were centrifuged for 5 min, 14,000 × g at 4°C. After discarding the supernatant, the remaining pellet was washed with ice-cold acetone and centrifuged for 5 min, 14,000 × g at 4°C. The supernatant was discarded and 100 μl of boiling 1 × sample buffer (4 × sample buffer: 125 mM Tris-HCl (Sigma Aldrich, St. Louis, MO, USA), 20% glycerol (Sigma Aldrich, St. Louis, MO, USA), 4% SDS (Merck Millipore, Billerica, MA, USA), 100 mM DTT (Roche Diagnostics GmbH, Risch, Switzerland), bromphenol blue (Sigma Aldrich, St. Louis, MO, USA); ad 200 mL distilled water, pH 7.8, 1 × sample buffer: 100 μl PhosphoSTOP, 100 μl complete mini (both from Roche Diagnostics GmbH, Risch, Switzerland), 250 μl 4x sample buffer, 550 μl distilled water) was added, re-suspended and boiled for 7 min 100°C. This was followed by centrifugation for 4 min 14,000 × g at RT. The remaining supernatants of each sample were collected in fresh Eppendorf-tubes, and directly subjected to SDS-PAGE. Separated proteins were transferred from gels onto nitrocellulose membranes (15 min) using the BioRad turbo blotting device. Non-specific binding of antibodies was prevented by incubating the membranes for 1 h in blocking buffer (5% BSA in 100 mL T-TBS) at RT while shaking. To analyze citrullinated histone H3, the polyclonal rabbit anti-human histone H3 (citrulline R2+R8+R17) (Abcam, Cambridge, UK, cat. no.:ab5103) was diluted in blocking buffer (5% BSA in 100 mL T-TBS) and the membrane was incubated over night at 4°C, while gently shaking. The day after, the membrane was washed three times with T-TBS for 10 min and subsequently incubated with an anti-rabbit IgG, HRP-linked secondary antibody (Cell Signaling Technology, MA, USA) diluted in blocking buffer for 1 h, at RT while shaking. Afterwards, the membrane was washed three times with T-TBS for 10 min. Visualization of proteins was done by oxidation of a luminol-based substrate. Chemiluminesecence was detected using a chemiluminescence imager, and the software Fusion. Analysis of the protein bands was performed using ImageJ.

### Statistical Analysis

Statistical analysis was performed with Graph pad prism 6 Software. Data were analyzed using one way ANOVA or Student's *t*-test.

## Results

### Cdc42 Is a Negative Regulator of NET Formation in Primary Human Neutrophils

**Figure 1:**
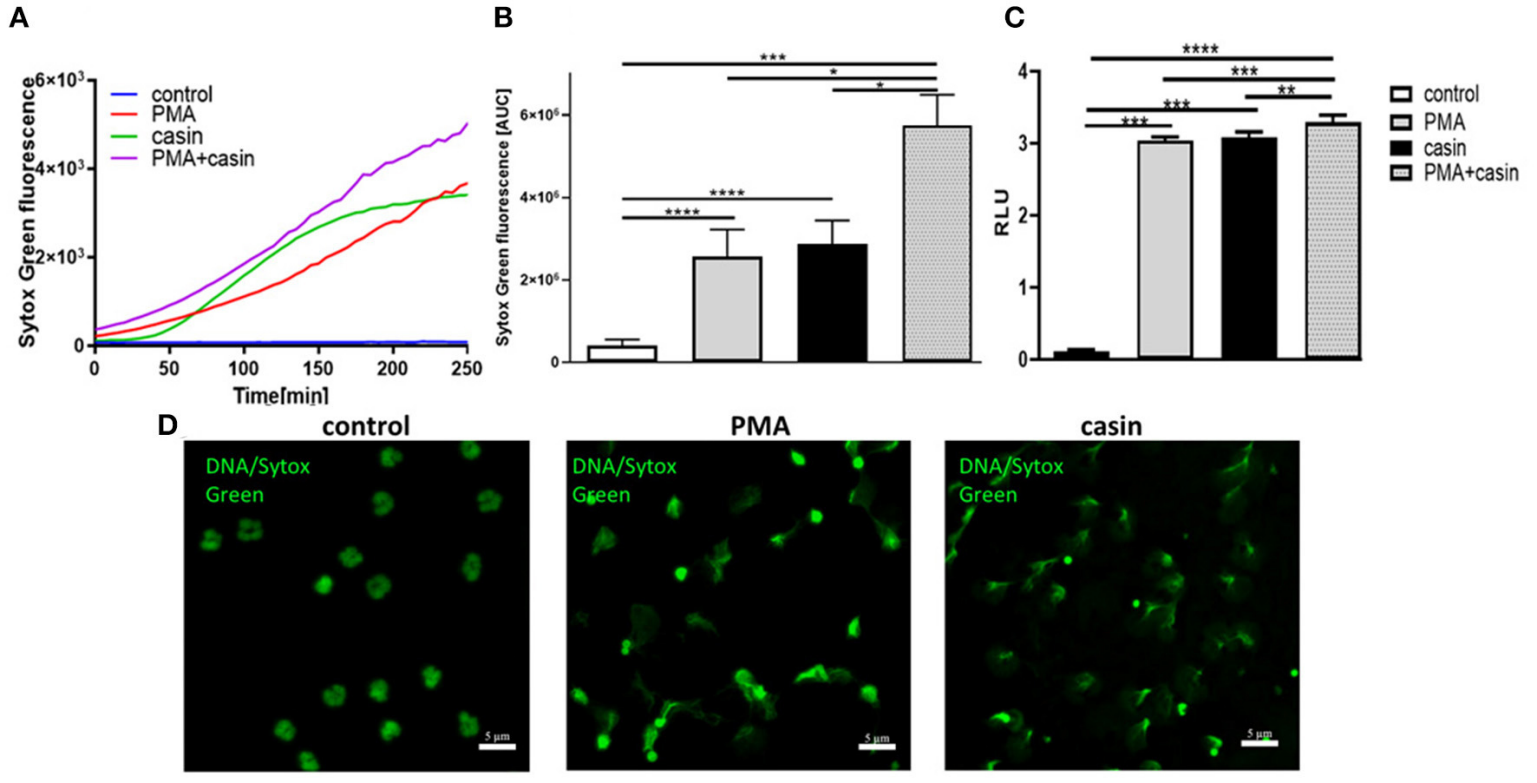
Cdc42 is a negative regulator of NET formation. Freshly isolated human neutrophils were seeded into a fluotrac 96-well plate and 5 μM Sytox Green was subsequently added to all samples. The formation of NETs without stimulation, with 20 nM PMA, with the Cdc42 inhibitor casin (10 μM) or a combination of PMA (20 nM) plus casin (10 μM) was detected over a period of 4 h at 37°C by measuring the fluorescence of DNA bound Sytox Green or by taking supernatants and performing an MPO-bound DNA ELISA. **(A)** Representative graph showing the Sytox Green fluorescence over time. **(B)** Quantification of NET release by calculating the area under the curve (AUC) from Sytox Green kinetic curves. **(C)** Quantification of NET release by measuring the OD at 405 nm of MPO-bound DNA, displayed as relative light units (RLU). Data show mean ± SD of 8 independent experiments. ****p ≤ 0.0001, ***p ≤ 0.001, **p ≤ 0.01, and *p ≤ 0.05. **(D)** Visualization of DNA bound Sytox Green using fluorescence microscopy.

The formation of NETs is a major antimicrobial effector mechanism of neutrophil granulocytes to efficiently trap and kill pathogens. To elucidate the role of Cdc42 activity in the formation of NETs, neutrophil granulocytes were treated with PMA or with the Cdc42 inhibitor casin or a combination of PMA and casin. Formation of NETs was analyzed by quantification of MPO-bound DNA in culture supernatants. To assess the kinetic of NET-release, fluorescence of the DNA intercalating dye SYTOX green was measured over time. The treatment with PMA is strongly inducing NET formation as shown by SYTOX green assay, ELISA as well as fluorescence staining (Figures 1A–D). Surprisingly, the inhibition of Cdc42 also induced the release of DNA (Figures 1A–D), which was confirmed by fluorescence microscopy (Figure 1D). Incubation of neutrophils with both, PMA and the Cdc42 inhibitor casin, increased NET formation compared to control cells, as well as in comparison to PMA or casin treated cells (Figures 1A–C).

### Cdc42 Inhibition Induces NETs in a Protein Kinase C Dependent Manner

**Figure 2:**
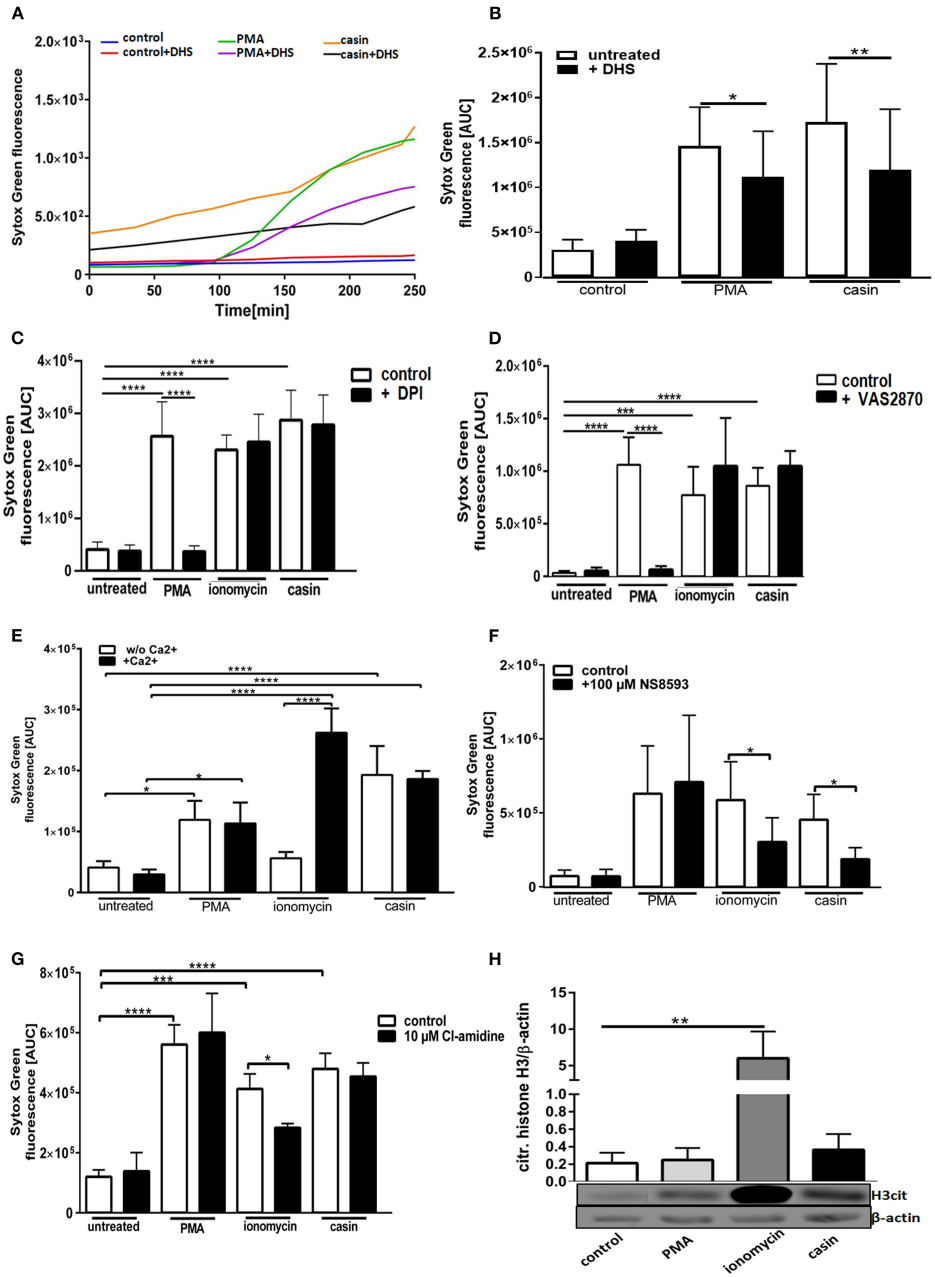
Cdc42 inhibition induces NETs in a Protein Kinase C and SK-channel dependent manner but independent of the NADPH oxidase, extracellular calcium, PAD4, and histone H3 citrullination. Freshly isolated human neutrophils were incubated with or without the Protein Kinase C inhibitor dihydrosphingosine (DHS) (4 μM) for 30 min at 37°C and subsequently 5 μM Sytox Green was added to all samples. The formation of NETs without stimulation (control), with 20 nM PMA or with the Cdc42 inhibitor casin (10 μM) was detected over a period of 4 h at 37°C by measuring the fluorescence of DNA bound Sytox Green **(A)**. NET release was quantified by calculating the area under the curve (AUC) from Sytox Green kinetic curves **(B)**. Data show mean ± SD of 7 independent experiments. **p ≤ 0.01 and *p ≤ 0.05. Freshly isolated human neutrophils were pre-incubated with the NADPH oxidase inhibitor DPI (20 μM) **(C)** or VAS2870 (20 μM) **(D)** for 30 min at 37°C, and 5 μM Sytox Green was subsequently added to all samples. The formation of NETs without stimulation (control), with 20 nM PMA, 10 μM ionomycin or with the Cdc42 inhibitor casin (10 μM) was detected over a period of 4 h at 37°C by measuring the fluorescence of DNA bound Sytox Green. Quantification of NET release by calculating the area under the curve (AUC) from Sytox Green kinetic curves. Data show mean ± SD of 5 independent experiments. ****p ≤ 0.0001 and ***p ≤ 0.001. Freshly isolated human neutrophils were re-suspended in Ca^2+^ deficient or Ca^2+^ containing medium **(E)** or pre-incubated with the SK-channel inhibitor NS8593 for 30 min at 37°C **(F)** and subsequently 5 μM Sytox Green was added to all samples. The formation of NETs without stimulation (untreated), with 20 nM PMA, 10 μM ionomycin or with the Cdc42 inhibitor casin (10 μM) was detected over a period of 4 h at 37°C by measuring the fluorescence of DNA bound Sytox Green. Quantification of NET release by calculating the area under the curve (AUC) from Sytox Green kinetic curves. Data show mean ± SD of 3–5 independent experiments. ****p ≤ 0.0001 and *p ≤ 0.05. Freshly isolated human neutrophils were pre-incubated with the PAD4 inhibitor Cl-amidine for 30 min at 37°C and subsequently 5 μM Sytox Green was added to all samples. The formation of NETs without stimulation (untreated), with 20 nM PMA, 10 μM ionomycin or with the Cdc42 inhibitor casin (10 μM) was detected over a period of 4 h at 37°C by measuring the fluorescence of DNA bound Sytox Green. **(G)** Quantification of NET release by calculating the area under the curve (AUC) from Sytox Green kinetic curves. **(H)** Freshly isolated human neutrophils were stimulated for 1 h at 37°C with 20 nM PMA, 10 μM ionomycin or 10 μM casin and the amount of citrullinated histone H3 was analyzed by western blotting, and normalized to β-actin. Data show mean ± SD of 3 independent experiments. ****p ≤ 0.0001, ***p ≤ 0.001, **p ≤ 0.01, and *p ≤ 0.05.

**Figure 3:**
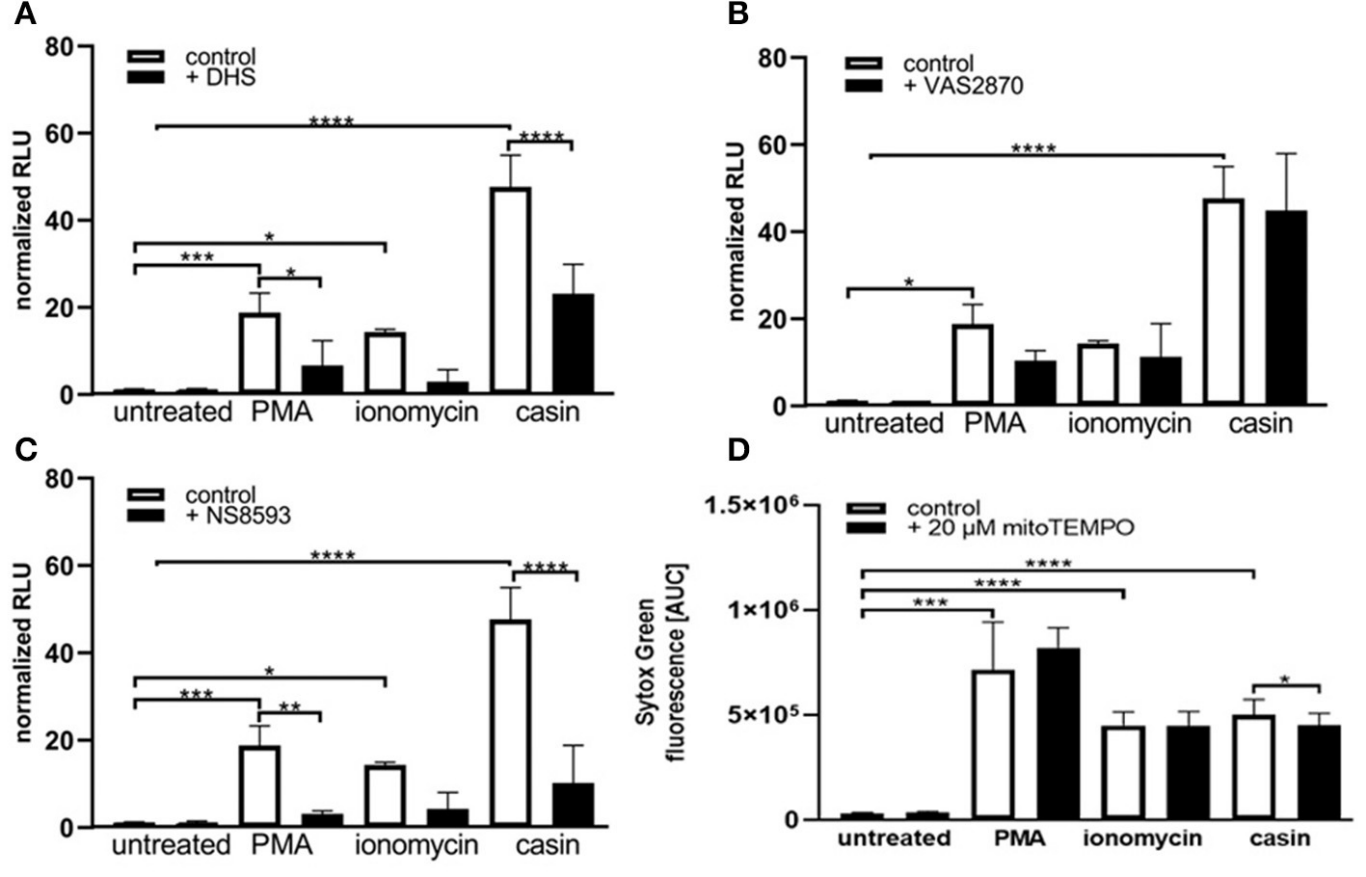
Cdc42 inhibition induces NET formation dependent on PKC, SK-channels and mitochondrial ROS. Freshly isolated human neutrophils were pre-incubated with the Protein kinase C inhibitor DHS (4 μM) **(A)**, the NADPH oxidase inhibitor VAS2870 (20 μM) **(B)** or the SK-channel inhibitor NS8593 (10 μM) **(C)** for 4 h at 37°C. The formation of NETs without stimulation (control), with 20 nM PMA, 10 μM ionomycin or with the Cdc42 inhibitor casin (10 μM) was quantified by measuring the OD at 405 nm of MPO-bound DNA, displayed as normalized relative light units (RLU). Data show mean ± SD of 3 independent experiments. ****p ≤ 0.0001, ***p ≤ 0.001, **p ≤ 0.01, and *p ≤ 0.05. **(D)** Freshly isolated human neutrophils were pre-incubated with the mitochondrial ROS inhibitor mitoTEMPO (20 μM) for 30 min at 37°C and seeded into a fluotrac 96-well plate. Subsequently, 5 μM Sytox Green was added to all samples and the formation of NETs without stimulation (control), with 20 nM PMA, 10 μM ionomycin or with the Cdc42 inhibitor casin (10 μM) was detected over a period of 4 h at 37°C by measuring the fluorescence of DNA bound Sytox Green. Quantification of NET release by calculating the area under the curve (AUC) from Sytox Green kinetic curves. Data show mean ± SD of 6 independent experiments. ****p ≤ 0.0001, ***p ≤ 0.001, and *p ≤ 0.05.

We next sought to understand the mechanism of Cdc42 inhibition-induced NETs. The PMA-induced NET-formation is dependent on ROS production via the NOX-complex and protein kinase C (PKC) (33). We thus first investigated the role of PKC in this Cdc42-dependent process. In the presence of the PKC inhibitor dihydrosphingosine (DHS), NETs formed in response to PMA were significantly reduced (Figures 2A,B, 3A). Furthermore, we observed that the DNA release in response to Cdc42 inhibition was also significantly reduced by inhibiting PKC (Figures 2A,B, 3A). In contrast, inhibition of PKC did not significantly reduce the ionomycin induced NET formation (Figure 3A).

### Cdc42 Inhibition Induces NETosis Through a NADPH-Oxidase Independent Pathway

The generation of ROS by the NADPH-oxidase is a hallmark of PMA induced NETosis (4, 33–35). However, it is known that stimuli like LPS, IL-8 or the calcium ionophore ionomycin can induce NETosis without the involvement of NOX activity (4, 5, 33–35). To test whether Cdc42 inhibition induces NET formation in a NOX-dependent or NOX-independent manner, primary human neutrophils were treated with the NOX inhibitors DPI and VAS2870 and the kinetics of NET-release was measured. Consistent with the literature, PMA-induced NET formation was dependent on NOX, shown by significantly reduced NET-extrusion in the presence of NOX inhibitors (Figures 2C,D and Supplementary Figure 1). As expected, the formation of NETs in response to the calcium ionophore ionomycin was not influenced by inhibition of NOX. Interestingly, the NET formation in response to Cdc42 inhibition was not changed when cells were treated with the NOX inhibitors (Figures 2C,D). The formation of NETs by NOX-inhibited human neutrophils in response to PMA, ionomycin and Cdc42 inhibition was also tested by quantification of MPO-bound DNA. Inhibition of NOX by VAS2870 only slightly, but not significantly reduced NET formation in response to PMA, while showing no effect whatsoever in ionomycin treated and Cdc42 inhibited cells (Figure 3B). This data indicates that PMA is inducing NETs via a NOX-dependent pathway, while ionomycin and Cdc42 inhibition induce NETs in a NOX-independent manner.

### Cdc42 Inhibition Induces NET Formation Independent on Extracellular Calcium but Dependent on SK Channels

Calcium-activated potassium channels of small conductance (SK-channels) as well as the presence of extracellular calcium play a special role in the NOX-independent formation of NETs (4). PMA is a direct PKC activator which, in turn, leads to calcium fluxes within the cell and both processes are described to be important for PMA-induced NETosis (34). To characterize the role of calcium in NET formation, cells were kept either in Ca^2+^- free (Figure 2E, white bars) or Ca^2+^- containing (Figure 2E, black bars) medium and NETosis was induced with PMA, ionomycin, and Cdc42 inhibition. The formation of NETs in response to PMA was independent on calcium since NETs were equally formed in both media (Figure 2E). NETs stimulated by ionomycin were highly dependent on calcium as shown by significantly lower NET formation in calcium-free medium compared to calcium-containing medium (Figure 2E). In contrast to ionomycin, the formation of NETs in response to Cdc42 inhibition was not altered in calcium-containing medium. This data indicates that PMA stimulation and Cdc42 inhibition induces NETs independent on extracellular calcium, thus implying that intracellular calcium stores may be sufficient for this process.

SK-channels are the major calcium-activated potassium channels known to be present on neutrophils (4). Neutrophils exhibit a potassium current which is activated by calcium influx (4). Ionomycin induces NET formation mediated by SK-channels while PMA induced NETosis was described to be independent of these channels (4). To elucidate the SK-channel dependency of PMA, ionomycin, and Cdc42 inhibition induced NETosis human neutrophil granulocytes were treated with the SK-channel inhibitor NS8593. NET release was induced by adding PMA, ionomycin, or the Cdc42 inhibitor casin. We showed here that SK-channels did not alter NET formation in response to PMA, as measured by analyzing Sytox green kinetics as indicator for NETosis (Figure 2F). In contrast, when we measured MPO-bound DNA in culture supernatants of neutrophils treated with the SK-channel inhibitor and subsequent PMA stimulation, we could show that PMA induced NETosis is indeed dependent on SK-channel activity (Figure 3C). Similarly we showed here that NET formation in response to Cdc42 inhibition, like ionomycin, was significantly reduced when SK-channels were inhibited (Figures 2F, 3C). This data indicates that extracellular calcium seems to be not important for NET formation in response to PMA and Cdc42 inhibition, while being a prerequisite for ionomycin induced NETosis. Furthermore, this data highlights that SK-channel activity is necessary for NET formation induced by PMA, ionomycin and Cdc42 inhibition.

### Cdc42 Inhibition Induces NET Formation Independent on PAD4 and Histone H3 Citrullination

It has been described that NOX independent formation of NETs requires an increase in intracellular calcium concentration which can be induced by calcium ionophores (3). An increase in cytoplasmic calcium leads to PAD4-complex formation with calcium (3). The formed complex translocates to the nucleus where it deaminates positively charged arginine, present on histones into non-charged citrulline (3). Citrullination of histones is considered to be one of the drivers of DNA decondensation and NETosis (3). To investigate the role of PAD4 in NETosis induced by Cdc42-inhibition, we treated primary human neutrophils with the irreversible PAD4 inhibitor Cl-amidine. NET formation was induced by adding PMA, ionomycin or the Cdc42 inhibitor casin. We could show that Cdc42-inhibition-induced NET release, like PMA induced NET formation were independent on PAD4, indicated by equal formation of NETs by cells treated with or without the PAD4 inhibitor (Figure 2G). In contrast, NET formation induced by ionomycin was partially dependent on PAD4 (Figure 2G).

Histone citrullination is a hallmark of NOX-independent NETosis but not NOX-dependent NETosis (3). Ionophore-mediated, but not PMA-mediated NETosis induces extensive citrullination of histone H3 (3). To test whether Cdc42 inhibition induces hypercitrullination of histones, western blot analysis of citrullinated H3 was performed. In accordance with the data from other groups, PMA did not induce citrullination of H3 while ionomcyin caused a significantly increased H3 citrullination (Figure 2H). The inhibition of Cdc42, similar to PMA, did not induce a hypercitrullination of histone H3 (Figure 2H). This data indicates that Cdc42 inhibition induces NET formation in a PAD4 independent mechanism which is not characterized by hypercitrullination of histones.

### Cdc42 Inhibition Induces NET Formation Dependent on Mitochondrial ROS

As shown in Figures 2F, 3C, SK-channels are involved in the formation of NETs in response to Cdc42-inhibition. SK-channels are known to activate mitochondrial ROS (mtROS) formation (4). It is assumed that mature human neutrophils contain very few mitochondria since they normally rely on glycolysis for energy production (36). However, Fossati et al. showed that freshly isolated neutrophils harbor an intricate network of mitochondria, which are a major source of ROS (4, 36). It was recently shown that PMA-stimulated neutrophils did not produce substantial amounts of mtROS (4, 5), whereas calcium ionophores triggered significantly higher amounts of mtROS, as measured by mitoSOX dye (4). This is consistent with the observation that NOX-independent stimuli of NETosis rely on mtROS for NETosis (4). To investigate the influence of mtROS on NET formation induced by PMA, ionomycin and Cdc42-inhibition, we used the mitochondria-restricted antioxidant mitoTEMPO to specifically inhibit mtROS. MitoTEMPO was described to act mitochondria specific to scavenge superoxide, thereby preventing mitochondrial damage (37, 38). We could show that NET formation induced by PMA or ionomycin cannot be dampened by inhibiting mtROS formation (Figure 3D). However, the inhibition of mtROS significantly, albeit only slightly, reduced the NET formation in response to Cdc42-inhibition (Figure 3D). This data suggests that the NET release in response to Cdc42-inhibition is in part caused by mtROS, which is not the case for NETs formed in response to PMA or ionomycin.

Since NETs induced by Cdc42 inhibition appear to have a specific dependency on mitochondrial ROS, we explored the role of mitochondria in more detail.

**Figure 4:**
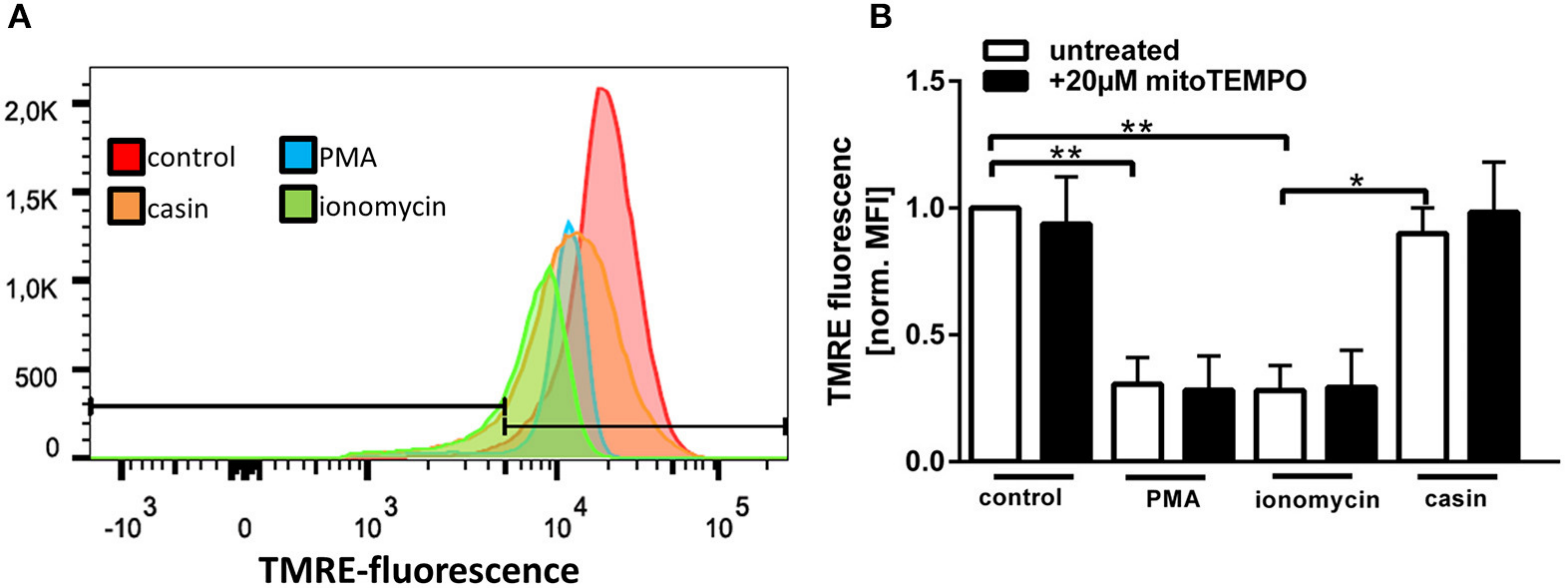
Cdc42 inhibition does not change the mitochondrial membrane potential. Freshly isolated human neutrophils were pre-incubated with or without the mitochondrial ROS inhibitor mitoTEMPO (20 μM) for 30 min at 37°C. Subsequently, cells were left untreated (control) or treated for 1 h with 20 nM PMA, 10 μM ionomycin or with the Cdc42 inhibitor casin (10 μM) at 37°C. Neutrophils were incubated with the mitochondrial membrane potential indicator TMRE for 30 min at 37°C followed by flow cytometry analysis. **(A)** Representative histogram of TMRE fluorescence. **(B)** Displayed is the mitochondrial membrane potential normalized to the value of control cells, in response to different stimuli with or without the inhibition of mitochondrial ROS. Data show mean ± SD of 3 independent experiments. **p ≤ 0.01 and *p ≤ 0.05.

Maintenance of a stable mitochondrial membrane potential (MMP) is a prerequisite of normal cell functions (32). The membrane potential is generated by proton pumps, namely the complexes I, III, and IV of the mitochondrial respiratory chain and is regulated by calcium (32). When the MMP is high, the respiratory chain becomes a significant producer of ROS (32). We thus sought to analyze the MMP by TMRE staining (Supplementary Figure 2). TMRE is a cationic fluorophore, which accumulates in polarized mitochondria in a concentration dependent manner that is directly related to the mitochondrial membrane potential (39, 40). Upon accumulation, TMRE exhibits a red shift in both its absorption and fluorescence emission spectra which can be measured using flow cytometry. Several groups described that functional, polarized mitochondria accumulate the positively charged dye in direct relation to the negative membrane potential within the mitochondria (39, 40). If the MMP is increasingly lost, the fluorescence signal of TMRE dissipates as the MMP decreases (39). Therefore, TMRE was used here as an indicator for changes in the MMP. Treatment of human PMN with PMA or ionomycin led to a significant reduction of the MMP (Figures 4A,B). Inhibition of Cdc42, however, did not significantly reduce the MMP compared to untreated cells. The MMP of Cdc42 inhibited cells was also higher than that of cells treated with ionomycin (Figures 4A,B). This data indicates that PMA and ionomycin, but not the inhibition of Cdc42 results in a strong depolarization of mitochondria.

Since MMP and mtROS production correlate with each other, we next investigated if mtROS is the reason for changes in the MMP, by using the mitochondria-targeting antioxidant mitoTEMPO. MMP changes in response to PMA, ionomycin and Cdc42 inhibition were not altered by mitoTEMPO, indicating that the changes of the MMP are not dependent on the production of mtROS (Figure 4B). Together, the data suggests that maintaining MMP and thus high levels of mtROS may be a mechanism by which Cdc42 inhibition enhances subsequent NET formation.

### Inhibition of Mitochondrial Respiratory Chain Complexes I and III Increase the Production of Mitochondrial ROS Which Is Related to NET Formation

**Figure 5:**
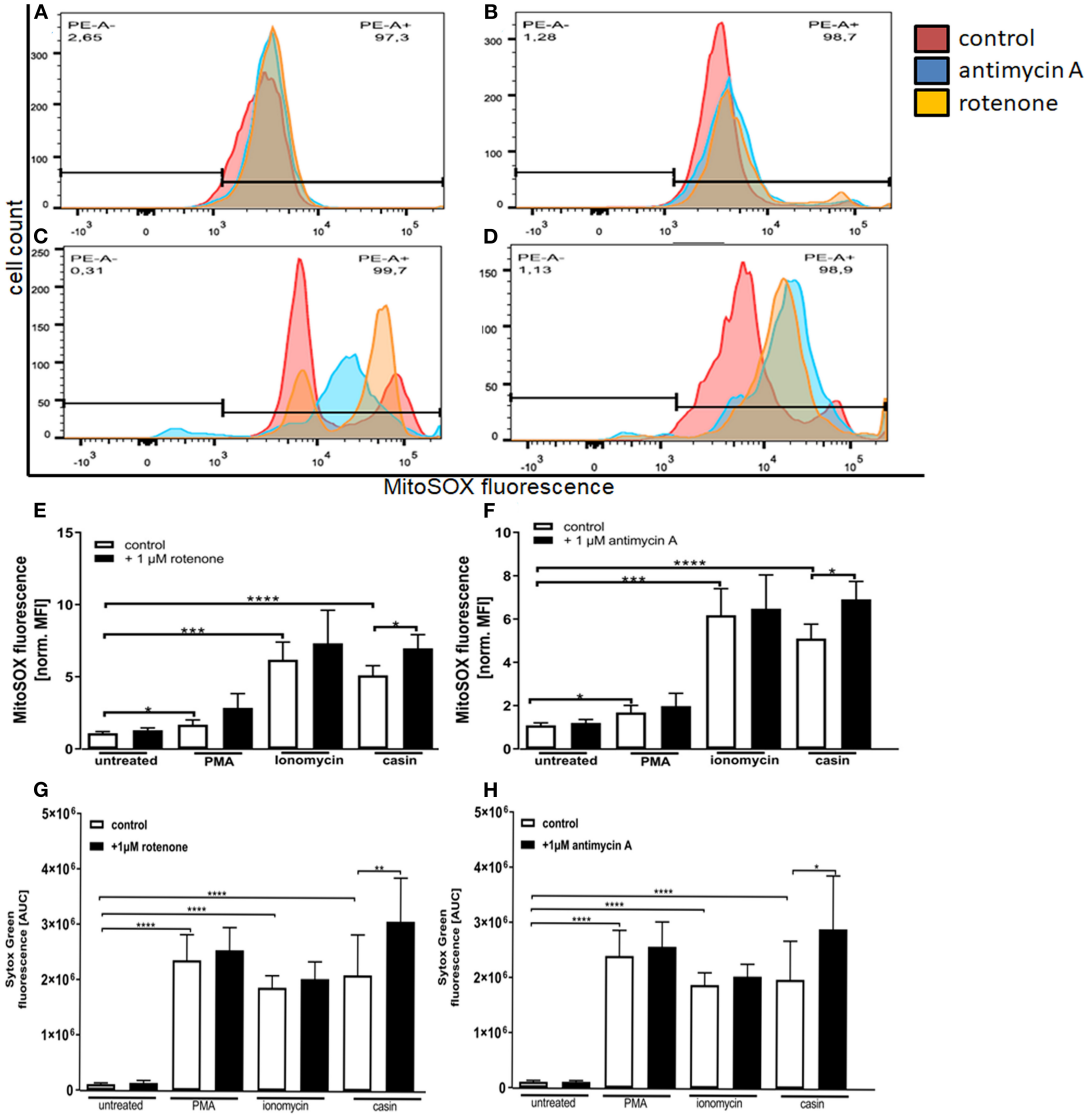
Inhibition of mitochondrial respiratory chain complexes I and III increase the production of mitochondrial ROS which is related to NET formation. Freshly isolated primary human neutrophils were left untreated or pre-incubated with 1 μM antimycin A **(A–D,F)** or 1 μM rotenone **(A–D,E)** for 30 min at 37°C. Subsequently, cells were incubated with 5 μM MitoSOX for 20 min at 37°C. Cells were either left unstimulated or stimulated with PMA (20 nM), ionomycin (10 μM) or the Cdc42 inhibitor casin (10 μM) for 1 h at 37°C. The fluorescence signal of MitoSOX was analyzed using flow cytometry. Displayed are representative histograms showing the MitoSOX fluorescence of control cells **(A)** and cells stimulated with PMA **(B)**, ionomycin **(C)**, or the Cdc42 inhibitor casin **(D**). **(E,F)** Quantification of MitoSOX mean fluorescence (MFI) normalized to the MitoSOX MFI of untreated cells. Data show mean ± SD of 4 independent experiments. ****p ≤ 0.0001, ***p ≤ 0.001, **p ≤ 0.01, and *p ≤ 0.05. Freshly isolated human neutrophils were pre-incubated with or without 1 μM rotenone **(G)** or 1 μM antimycin A **(H)** for 30 min at 37°C, followed by incubation with or without the mitochondrial ROS indicator MitoSOX (5 μM) for 20 min at 37°C. The formation of NETs without stimulation (untreated), with 20 nM PMA, 10 μM ionomycin or with the Cdc42 inhibitor casin (10 μM) was detected over a period of 4 h at 37°C by measuring the fluorescence of DNA bound Sytox Green. Quantification of NET release by calculating the area under the curve (AUC) from Sytox Green kinetic curves. Data show mean ± SD of 3 independent experiments. ****p ≤ 0.0001, **p ≤ 0.01, and *p ≤ 0.05.

As previously described, the MMP is generated by the respiratory chain complexes I, –III, and –IV (32). Complex IV is not as essential for this process, since neutrophil mitochondria contain very little cytochrome c, which is an essential component of complex IV (31). The inhibition of both complexes was shown to significantly up-regulate the formation of mtROS in neutrophils isolated from mice with an inactive NADPH-oxidase (41). Based on this, we next tested the influence of complex I and –III inhibition on the production of mtROS and on NET formation. We could show that incubation of human neutrophils with PMA, ionomycin or the Cdc42 inhibitor casin induces a significant increase in mitochondrial ROS production (Figures 5A–H). When cells were pre-incubated with the complex I and complex III inhibitors rotenone and antimycin A, respectively, Cdc42-inhibited cells showed increased mtROS production, compared to cells tested without previous addition of inhibitors (Figures 5D–F). In contrast, the inhibition of complex I and III did not further increase the production of mtROS in response to PMA and ionomycin (Figures 5B,C,E,F). Based on this data and the theory that high mtROS causes NET formation in Cdc42 inhibition mediated NETosis, NET-release in response to complex I and III inhibition was analyzed. Inhibition of both complexes did not change the formation of NETs by untreated cells (Figures 5G,H). Furthermore, NET formation in response to PMA or ionomycin was not altered due to complex I or III inhibition, although a slight increase of NET-release could be observed in both conditions (Figures 5G,H). In contrast, the formation of NETs in response to Cdc42 inhibition was significantly increased when human neutrophils were treated with the complex I and III inhibitors rotenone and antimycin A, respectively (Figures 5G,H). This data supports the theory that Cdc42 inhibition is engaging the mitochondrial respiratory chain and that NET formation due to Cdc42 inhibition depends on mtROS.

### Cdc42 Deficient Murine Neutrophils Show Increased NET Formation Which Is Independent of the NADPH-Oxidase

**Figure 6:**
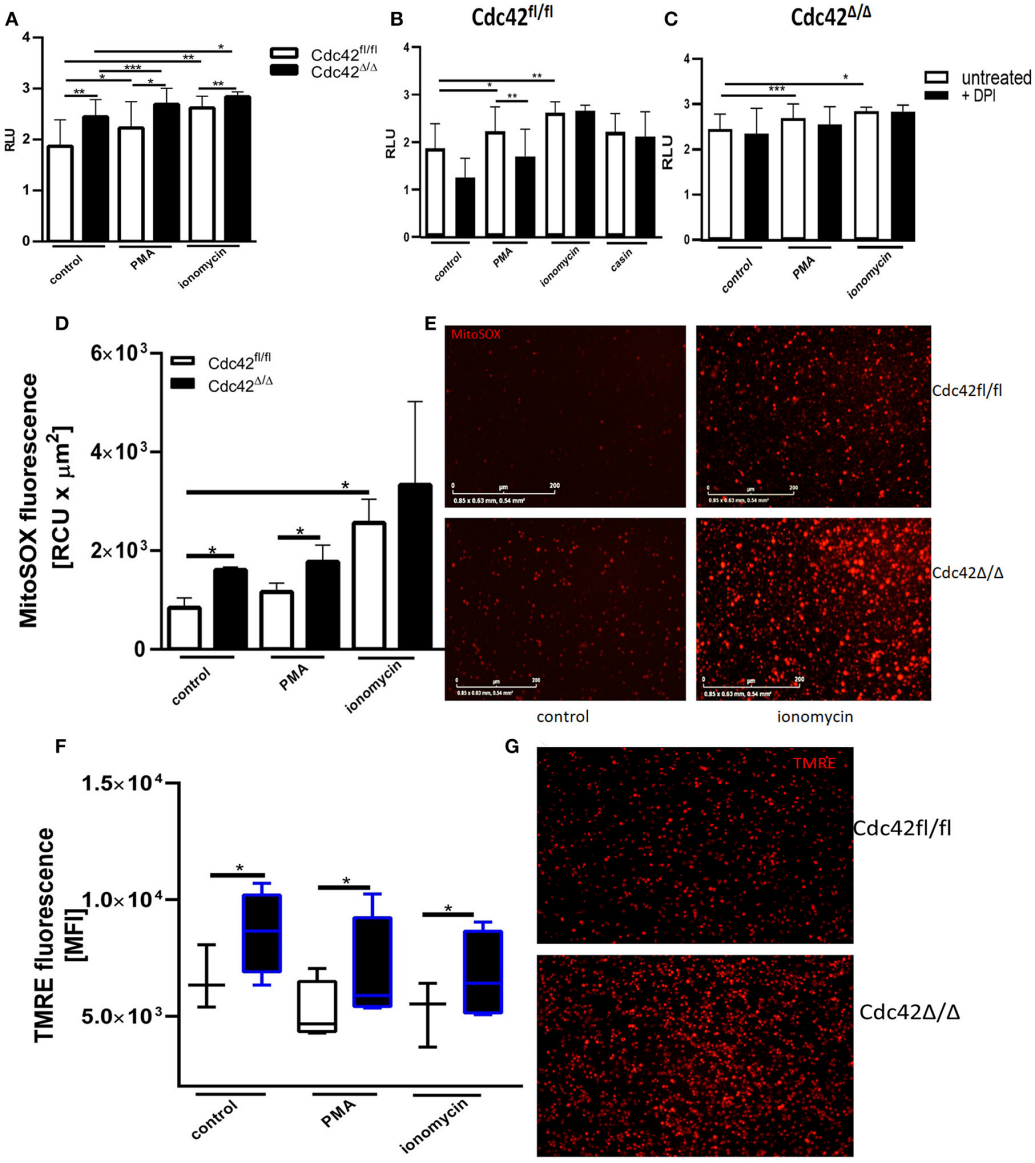
Cdc42 deficient murine neutrophils show increased NET formation in a NADPH oxidase independent manner and display enhanced mitochondrial membrane potential and mitochondrial ROS production. **(A)** Freshly isolated bone marrow derived murine neutrophils from Cdc42^fl/fl^ and Cdc42^Δ/Δ^ mice were left untreated (control) or stimulated with 100 nM PMA or 5 μM ionomycin for 3 h at 37°C. Quantification of NET release by measuring the OD at 450 nm of histone-bound DNA, displayed as relative light units (RLU). Data show mean ± SD of 8 independent experiments. ***p ≤ 0.001, **p ≤ 0.01, and *p ≤ 0.05. **(B,C)** Freshly isolated bone marrow derived murine neutrophils from Cdc42^fl/fl^ and Cdc42^Δ/Δ^ mice were pre-incubated with the NADPH-oxidase inhibitor DPI for 30 min at 37°C. Subsequently, cells were left untreated or stimulated with 100 nM PMA, 5 μM ionomycin or the Cdc42 inhibitor casin (10 μM, Cdc42^fl/fl^ only) for 3 h at 37°C. Quantification of NET release by measuring the OD at 450 nm of Histone-bound DNA, displayed as relative light units (RLU). Data show mean ± SD of 4 independent experiments. ***p ≤ 0.001, **p ≤ 0.01, and *p ≤ 0.05. **(D,E)** Freshly isolated bone marrow derived murine neutrophils from Cdc42^fl/fl^ and Cdc42^Δ/Δ^ mice were seeded into a 96-well glass bottom plate. Subsequently, cells were incubated with the mitochondrial ROS indicator mitoSOX for 20 min at 37°C. The formation of mitochondrial ROS of untreated cells (control) or in response to 100 nM PMA, or 5 μM ionomycin was detected for 1 h, every 4 min at 37°C using the IncuCyte life imaging. **(D)** Quantification of mitochondrial ROS by calculating the relative fluorescence of mitoSOX. Data show mean ± SD of 3 independent experiments *p ≤ 0.05. **(E)** Representative images of the mitoSOX signal of control and ionomycin treated Cdc42^fl/fl^ and Cdc42^Δ/Δ^ cells. Freshly isolated bone marrow derived murine neutrophils from Cdc42^fl/fl^ and Cdc42^Δ/Δ^ mice were left untreated (control) or stimulated with 100 nM PMA or 5 μM ionomycin. Subsequently, cells were incubated with the mitochondrial membrane potential indicator TMRE for 30 min at 37°C. **(F)** Mitochondrial membrane potential in response to different stimuli assessed by using flow cytometry. Data show mean ± SD of 5 independent experiments *p ≤ 0.05. **(G)** Representative images showing the TMRE signal of untreated Cdc42^fl/fl^ and Cdc42^Δ/Δ^ cells.

To further assess the effect of Cdc42-inhibition on NET formation, we used bone marrow neutrophils from Cdc42^fl/fl^ (considered as wild type) and Cdc42^Δ/Δ^ (considered as Cdc42 knock out) mice. The formation of spontaneous NETs or in response to PMA and ionomycin was analyzed by measuring the amount of histone-bound DNA. Cdc42-deficient neutrophils show significantly higher spontaneous as well as PMA- and ionomycin-induced NET formation compared to cells from wild type mice (Figure 6A). This data indicates that the earlier observations in human primary neutrophils were not induced by off-target effects of the inhibitor casin but rather display a direct effect of disrupted Cdc42 activity.

Cdc42^fl/fl^ neutrophils produced significantly more NETs in response to PMA and ionomycin treatment (Figure 6B). However, only PMA-induced NET formation of Cdc42^fl/fl^ neutrophils was reduced upon inhibition of the NADPH-oxidase. Further, NETs formed by Cdc42^Δ/Δ^ neutrophils, like those formed in response to Cdc42 inhibition, were independent of NADPH-oxidase (Figure 6C). Cdc42^Δ/Δ^ neutrophils also displayed increased mtROS (Figures 6D,E) and maintained a high MMP (Figures 6F,G) while forming NETs.

## Discussion

Neutrophil Extracellular Traps (NETs) represent a major antimicrobial effector mechanism of neutrophil granulocytes due to their ability to trap and kill pathogens. It is known that the formation of NETs can be induced by many pathogens but also by sterile stimuli like cytokines, immune complexes, auto-antibodies and PMA or calcium ionophores (11). The production of ROS by the NOX-complex was implicated to be an essential event in NETosis (5, 11). However, it is now clear that stimulators of NOX, and therefore ROS production, are not sufficient to induce NET formation, and NETs can also form independently of ROS (5). Some stimuli like LPS, IL-8, or calcium-ionophores but also some pathogens trigger NETosis without engaging the NOX-complex (5). These observations highlight that NETosis can occur via NOX-dependent and NOX-independent pathways (4, 5). However, the mechanisms by which NOX-independent NETosis occurs are not well-understood (4). The sGTPase Cdc42 is mainly known to regulate cytoskeleton rearrangement. Here, using a pharmacological inhibitor of Cdc42 and a genetic model, we found that Cdc42 is a negative regulator of NET formation in a NOX-independent manner. We showed that inhibition of Cdc42 increases NET formation via PKC, SK-channel and mitochondrial activity. Since Cdc42 inhibition was shown to depend on PKC and SK-channel activity, it is possible that Cdc42 in its active state is normally suppressing the function of PKC and SK-channels. When the action of Cdc42 is inhibited, the suppressing effect on both might be blocked, and PKC as well as SK-channels are activated to induce NET formation.

NETs are known to be formed NOX-dependent or NOX-independent (4, 5, 33–35). Gray et al. showed that the NET forming activity of the diacylglycerol (DAG)-imitating compound PMA is critically dependent on PKC pathways upstream of NOX (33). Additionally they confirmed the key role of ROS production by the NOX-complex in PMA-induced NETosis (33). In this study, we show that Cdc42 inhibition induces the formation of NETs in primary human neutrophils in a PKC-dependent manner. Blocking of PKC is sufficient to significantly decrease NET formation in response to PMA and Cdc42 inhibition. However, while the NET forming activity of PMA was shown to be dependent on NOX-derived ROS (3, 4, 33, 42), Cdc42 inhibition induces NET formation independent of NOX and therefore independent of NOX-derived ROS. We proved this by showing that NOX inhibition results in reduced NET formation in response to PMA but not in response to ionomycin or Cdc42 inhibition. This is consistent with studies showing that although Cdc42 can bind to the NOX component gp91^phox^ *in vitro*, Cdc42 is unable to stimulate ROS formation by the NOX-complex (43).

Douda et al. presented evidence that SK-channels and extracellular calcium are prerequisites of NOX-independent NETosis (4). They showed that calcium ionophore-induced NETosis is mediated by SK-channels, and claimed that this is not the case for PMA-induced NET formation (4). The role of calcium in NET formation is still poorly understood. It is hypothesized that intracellular calcium concentration increases during early stages of NET formation (44). An increase in intracellular calcium, induced by calcium ionophores was described to be important for histone citrullination (3). Citrullination of histones is considered to be the driver of DNA decondensation and NETosis and is a hallmark of NOX-independent NETosis (3). Calcium is building a complex with PAD4, which translocates to the nucleus to deaminate positively charged arginine on histones into non-charged citrulline (3, 44). de Bont et al. demonstrated that PMA stimulation evokes a small transient change in cytosolic calcium concentration, followed by a gradual increase (44). This gradual increase seems to continue until the plasma membrane ruptures (44). In contrast, the same group demonstrated that calcium ionophores induce a single transient increase in cytosolic calcium concentration, localized mainly to intracellular foci such as vesicles, the endoplasmatic reticulum and granules, which are emptied out by ionophore action (44). Furthermore, the same group showed that the rapid increase in cytosolic calcium resulted in PAD activation and prominent histone H3 citrullination (44). In line with this observation, we showed a dependence of ionomycin induced NETosis on PAD4 and increased histone H3 citrullination. In contrast, we showed here that PAD4 inhibition does not reduce PMA or Cdc42 inhibition induced NET formation, and that PMA stimulation as well as Cdc42 inhibition does not result in histone H3 citrullination. Therefore, it is likely that the ionomycin induced cytosolic calcium increase activates PAD4, to induce histone H3 citrullination and NETosis, while PMA and Cdc42 inhibition seem to induce only marginal changes in cytosolic calcium concentration. This theory is based on the observation that neither PMA nor Cdc42 inhibition induced NET formation was reduced upon PAD4 inhibition and the fact that we did not observe changes in histone H3 citrullination when cells were treated with PMA or the Cdc42 inhibitor.

Here we show that Cdc42 inhibition-mediated NET formation is not dependent on extracellular calcium, like PMA induced NET release, in contrast to ionomycin-induced NET formation, which is dependent on calcium. Calcium oscillation and ROS production was described to coincide when cells were stimulated with PMA, implicating that calcium acts more as a second messenger for ROS production (44). PMA acts directly on PKC to induce ROS formation. Therefore, it is possible that we do not see any changes in PMA induced NETosis in either medium, because PKC is still activated by PMA to induce the formation of ROS, ultimately leading to NETosis. Furthermore, it seems likely that other processes like the production of mtROS drive NETosis induced by Cdc42 inhibition.

However, like ionomycin, Cdc42 inhibition- and PMA triggered NET release depends on SK-channel activity. This data indicates that Cdc42 inhibition and PMA stimulation induces NETosis without requiring additional extracellular calcium but requires SK-channel activity. This theory is also supported by data from other groups showing that chelation of extracellular calcium significantly decreases ionophore induced NETosis (4, 44, 45). This implies that intracellular calcium stores may be sufficient to activate SK-channels in cells treated with PMA and the Cdc42 inhibitor, while additional extracellular calcium is required for ionomycin induced NET formation.

Douda et al. linked the activity of SK-channels to mitochondrial ROS by showing that these channels activate mitochondrial ROS (4). The fact that DPI is not inhibiting NET formation in response to Cdc42 inhibition does not rule out the possibility that other ROS sources like mitochondrial ROS are involved in this process. Although it is assumed that mature human neutrophils possess very few functional mitochondria, Douda et al. showed significant mtROS production in response to calcium ionophores (4). They presented evidence that NET-independent NETosis relies on mtROS while no substantial mtROS formation could be observed in PMA-stimulated NOX-dependent NETosis (4). It is known that a major regulator of mtROS production is the mitochondrial membrane potential (MMP) (32). The superoxide anion is the most undesired by-product of mitochondrial oxidative phosphorylation (46). Its production is induced by leakage of electrons from the mitochondrial respiratory chain, and the reaction of these electrons with oxygen (46). Electrons can leak singly from the respiratory chain to molecular oxygen from 1-electron sites within the complexes I and III (47). In both complexes, the probability of electron leakage to form superoxide is increased at high MMP (47). At a low MMP, less time the electrons are delayed at the critical sites where leakage is possible (47). Zorova et al. showed that high MMP causes an increased production of mtROS by the mitochondrial respiratory chain. We showed here that Cdc42 deficient murine neutrophils produce significantly higher amounts of mtROS. Furthermore, we demonstrated that inhibition of mtROS reduces NET formation in response to Cdc42 inhibition in primary human neutrophils. This data is consistent with observations of Takishita et al. showing that mitoTEMPO did not completely suppress NET formation in cells stimulated with the calcium ionophore A23187 and PMA (37). This group hypothesized that not only mtROS but also mitochondrial signaling is involved in NET formation in response to PMA and the calcium ionophore (37).

We present evidence that Cdc42 deficient murine PMNs have a much higher MMP than wild-type neutrophils. This increased MMP may be causative for the higher mtROS production. We showed that stimulation of human neutrophils with PMA, ionomycin, but not Cdc42 inhibition, induces a depolarization of mitochondria, suggesting that maintenance of high MMP in response to Cdc42 inhibition may promote mtROS and subsequently higher NET release. Interestingly, excessive calcium influx normally results in transient depolarization of mitochondria (48). The lack of calcium influx in response to Cdc42 inhibition may help preserving MMP in this case.

The MMP is generated by the respiratory chain complexes I, –III, and –IV (32). Complex IV is not as essential for this process, since neutrophil mitochondria contain very little cytochrome c, which is an essential component of complex IV (31). The inhibition of both complexes was shown to significantly upregulate the formation of mtROS in neutrophils isolated from mice with an inactive NADPH-oxidase (41). Korshunov et al. showed that inhibition of complex I of the respiratory chain strongly increases ROS production in mitochondria, and demonstrated a very steep dependence of ROS production and the MMP, claiming that ROS formation is a function of MMP (47). Furthermore, the same group showed, that even a small increase in MMP gives rise to large stimulation of ROS production by mitochondria (47). We showed here that inhibition of complex I and –III increases the formation of mtROS, and additionally the formation of NETs in response to Cdc42 inhibition, while having no effect on PMA and ionomycin induced NET formation. It is described that the complex I inhibitor rotenone induces the mitochondrial complex I substrate-supported mtROS production (49). Rotenone blocks the mitochondrial respiratory chain complex I, thereby increasing the formation of semiubiquinone, the primary electron donor in mitochondrial superoxide generation, thereby increasing mtROS production (49). Similarly, antimycin A was described to enhance mtROS production by increasing the semiubiquinone levels within mitochondria (50). This led us to believe that inhibiting the mitochondrial respiratory chain plus Cdc42 activity promotes NET formation due to Cdc42 inhibition perhaps by increasing mtROS. The observation that inhibition of complex I and III only slightly, but not significantly increase the mtROS formation in cells that were stimulated with PMA or ionomycin suggests that different mitochondrial signaling events are involved in NETosis induced by Cdc42 inhibition and stimulation with PMA or ionomycin. Further investigation of the mitochondrial signaling events will be of great interest to elucidate differences between PMA, ionomycin and Cdc42 inhibition induced NET formation.

In summary, we present in this study a novel pathway of NET formation induced by Cdc42 inhibition or deficiency that differs from the already described PMA- or calcium ionophore induced NETosis. This pathway is independent on NOX but engages PKC. Furthermore, Cdc42 deficiency induces NETosis through activation of SK-channels and a high MMP. The MMP and the mitochondrial respiratory chain complexes I and III are likely responsible for increased mtROS production and subsequent NET formation. In this NETosis cascade, the activity of PAD4 and subsequent hypercitrullination of histone H3 does not play a role as it is the case for classic NOX-independent NETosis.

## Data Availability Statement

The raw data supporting the conclusions of this article will be made available by the authors, without undue reservation.

## Ethics Statement

The studies involving human participants were reviewed and approved by the ethical committee of the Medical Faculty of the University of Lübeck (18–187). The patients/participants provided their written informed consent to participate in this study. This animal study was conducted with a protocol approved by the Animal Care Committee of Cincinnati Children's Hospital Medical Center.

## Author Contributions

HT designed and performed experiments, analyzed data, and wrote the manuscript. SM assisted to perform experiments. M-DF and TL supported to design experiments, interpreted data, and revised the manuscript. All authors contributed to the article and approved the submitted version.

## Conflict of Interest

The authors declare that the research was conducted in the absence of any commercial or financial relationships that could be construed as a potential conflict of interest.
