# Supplementary material for: The Small GTPase Cdc42 Negatively Regulates the Formation of Neutrophil Extracellular Traps by Engaging Mitochondria
Source: Front Immunol. 2021 Feb 17;12:564720. doi: 10.3389/fimmu.2021.564720 (PMC7925625; doi:10.3389/fimmu.2021.564720)
Supplement: Supplementary file 1 [file Table_1.DOCX]

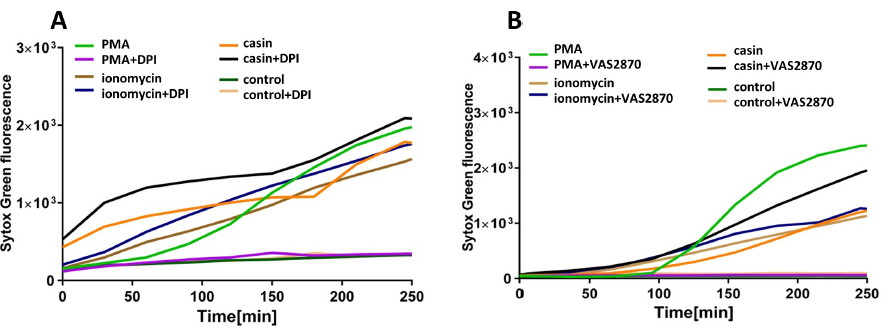


**Suppl. Fig. 1: Cdc42 inhibition induces NETs through a NADPH oxidase-independent pathway**

Freshly isolated neutrophils were pre-incubated with the NADPH oxidase inhibitor DPI (20 µM) **(A)** or VAS2870 (20 µM) **(B)** for 30 min at 37 °C. Subsequently 5 µM Sytox Green was added to all samples. The formation of NETs without stimulation, with 20 nM PMA, 10 µM ionomycin or with the Cdc42 inhibitor casin was detected over a period of 4 hrs at 37 °C by measuring the fluorescence of DNA bound Sytox Green. Representative kinetic curves of the Sytox Green fluorescence over time are displayed.

**
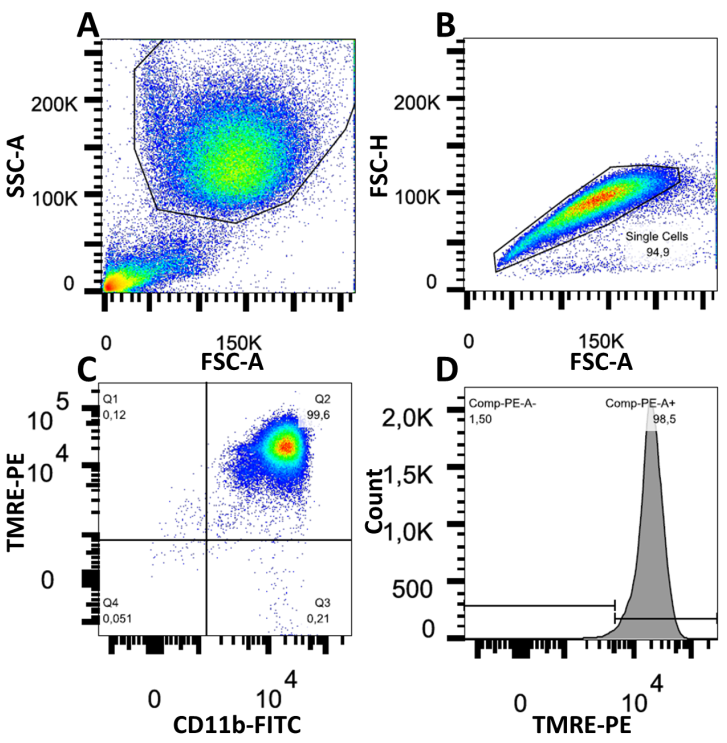
**

**Suppl. Fig. 2: Gating strategy to analyze the mitochondrial membrane potential**

Neutrophils were stained with an antibody to CD11b (FITC) and the mitochondrial membrane potential indicator TMRE (PE). Displayed are representative flow cytometry histograms of unstimulated PMNs. **(A)** Neutrophils were gated according to the size and granularity shown in the sideward- (SSC) and forward-scatter (FSC) dot plot. **(B)** Neutrophils were gated for single cells followed by **(C)** gating for TMRE-positive/CD11b positive cells in quadrant Q2. **(D)** Representative histogram showing the TMRE-mean fluorescence of CD11b positive cells.
